# Cannabis-Based Medicinal Products in the Treatment of Post-Traumatic Stress Disorder in Children and Young People: A Literature Review

**DOI:** 10.1192/bjo.2025.10171

**Published:** 2025-06-20

**Authors:** Qudus Lawal, Shatha Shibib, Adeola Animashaun

**Affiliations:** 1 Sheffield Children’s NHS Foundation Trust, Sheffield, United Kingdom; 2 County Durham and Darlington NHS Foundation Trust, Durham, United Kingdom

## Abstract

**Aims:** Post-traumatic stress disorder (PTSD) is a condition that may develop following exposure to a highly threatening or horrific event or series of events. PTSD can affect people across all age groups with extensive impact on functioning and is often associated with psychoactive substance misuse. Cannabis is one of the most abused psychoactive substances worldwide, with users reporting anxiolytic benefits. Cannabis-based medicinal products (CBMPs) have gained more attention and interest over the past few years due to changes in the legislation around cannabis worldwide. Research has shown cannabis-based medicinal products to be effective in treating several medical conditions. Observational studies in adult populations indicate some therapeutic promise for CBMPs in PTSD, but these results are not generalizable to younger populations.

The authors aimed to complete a search of the literature for any evidence of the benefit of cannabis-based medicinal products in treating children and young people diagnosed with PTSD.

**Methods:** A comprehensive search of databases, including Medline, Embase, PsycINFO, CINAHL, Cochrane Library, and Google Scholar, from their inception until September 2024, was conducted using medical subject headings and keywords: “Post-traumatic stress disorder”, “Medical Marijuana”, “Cannab*”, “Canab*”, “THC*”.

The authors limited their search to papers involving children and young people under 18 years of age. Three of the 105 papers screened – two systematic reviews and one case report – were eligible for inclusion in this review.

**Results:** The current evidence for treating post-traumatic stress disorder with cannabis-based medicinal products in children and young people under the age of 18 years is limited to case reports, making generalisability of the results to the general population difficult.

**Conclusion:** The evidence for the use of CBMPs in children and adolescents with PTSD is limited. While there are several studies based on adult participants suggesting the beneficial effects of CBMPs in PTSD, the results cannot be generalized. This review highlights the need for high-quality research to establish the efficacy and safety of CBMPs in treating PTSD among children and adolescents.

